# Leptin action in normal and pathological pregnancies

**DOI:** 10.1111/jcmm.13369

**Published:** 2017-11-21

**Authors:** Antonio Pérez‐Pérez, Ayelén Toro, Teresa Vilariño‐García, Julieta Maymó, Pilar Guadix, José L. Dueñas, Manuel Fernández‐Sánchez, Cecilia Varone, Víctor Sánchez‐Margalet

**Affiliations:** ^1^ Department of Medical Biochemistry and Molecular Biology Virgen Macarena University Hospital University of Seville Seville Spain; ^2^ Laboratory of Placental Molecular Physiology Department of Biological Chemistry School of Sciences University of Buenos Aires IQUIBICEN‐CONICET Buenos Aires Argentina; ^3^ Department of Obstetrics and Gynecology Virgen Macarena University Hospital University of Seville Seville Spain; ^4^ Valencian Infertility Institute (IVI) Seville Spain

**Keywords:** leptin, reproduction, placenta, polycystic ovary syndrome, recurrent miscarriage, pre‐eclampsia, gestational diabetes, growth restriction

## Abstract

Leptin is now considered an important signalling molecule of the reproductive system, as it regulates the production of gonadotrophins, the blastocyst formation and implantation, the normal placentation, as well as the foeto‐placental communication. Leptin is a peptide hormone secreted mainly by adipose tissue, and the placenta is the second leptin‐producing tissue in humans. Placental leptin is an important cytokine which regulates placental functions in an autocrine or paracrine manner. Leptin seems to play a crucial role during the first stages of pregnancy as it modulates critical processes such as proliferation, protein synthesis, invasion and apoptosis in placental cells. Furthermore, deregulation of leptin levels has been correlated with the pathogenesis of various disorders associated with reproduction and gestation, including polycystic ovary syndrome, recurrent miscarriage, gestational diabetes mellitus, pre‐eclampsia and intrauterine growth restriction. Due to the relevant incidence of the mentioned diseases and the importance of leptin, we decided to review the latest information available about leptin action in normal and pathological pregnancies to support the idea of leptin as an important factor and/or predictor of diverse disorders associated with reproduction and pregnancy.

**Table:** 

• Introduction • Leptin mediates the crosstalk between adipose tissue and reproduction • Role of leptin in placenta development • Leptin as an immunomodulator during pregnancy • Leptin and pathologies associated with pregnancy ‐ Polycystic ovary syndrome ‐ Recurrent miscarriage	‐ Gestational diabetes mellitus ‐ Pre‐eclampsia ‐ Intrauterine growth restriction • Conclusions • Acknowledgements • Conflicts of interests

## Introduction

Adipose tissue acts as an endocrine organ, secreting different molecules or adipokines 1. Leptin is produced and secreted predominantly from adipose tissue into the circulation. Circulating leptin levels reflect adipose tissue size and also change with nutritional state 2. Furthermore, leptin is considered as a pleiotropic hormone that regulates not only bodyweight but many other functions, including vascular function, bone and cartilage growth, immune system and systemic inflammatory response as well as the normal physiology of the reproductive system 3, 4.

A link between bodyweight, adipokines and success of pregnancy has been proposed, although it is not fully understood 5, 6, 7. The observations that human and rodents with congenital leptin deficiencies are sterile and that anorexia and obesity modify the onset of puberty in opposite ways, led to the idea that leptin is an important player in reproduction 8. In this way, leptin was the first adipokine claimed to be the ‘missing link’ between fat and reproduction 9.

Leptin mediates its effects by binding to leptin receptors (LepRs) expressed in the brain and peripheral tissues 2. Different variants of LepR have been described, but the long isoform of LepR (LepRb) is primarily responsible for leptin signalling. LepRb is strongly expressed in specific nuclei of the hypothalamus, a region of the brain that is involved in the control of appetite, and there it regulates energy homoeostasis and neuroendocrine function, among other functions 10. In addition, leptin has direct effects on many cell types on the periphery. LepRb is expressed in lung, kidney, adipocytes, endothelial cells, blood cells, stomach, muscle, liver, pancreatic islets, osteoblast, endometrium, placenta and umbilical cord 2, 11.

Leptin or LepR deficiencies not only cause severe obesity but also abnormalities in haematopoiesis, immunity, angiogenesis, bone formation, blood pressure and reproduction. Mutations in the leptin gene, in human and/or mouse models, result in infertility or significant reproductive dysfunction 8, 12. Leptin is required for the release of gonadotrophin‐releasing hormone (GnRH) from the pituitary, and as a consequence, female *ob/ob* mice (deficient in leptin) have reduced oestrogen levels and exhibit low uterine weight 13, 14. Male *ob/ob* mice also show reduced GnRH levels and diminished production of luteinizing hormone (LH) and follicle‐stimulating hormone (FSH) as well as testosterone, an essential hormone for the maintenance of male fertility and testicular function 15.

Therefore, leptin can act as metabolic switch connecting the nutritional status of the body to high energy consuming processes. The energy requirements of pregnancy are those desired for correct maternal gain to ensure the growth of the foetus, placenta and associated maternal tissues 12. Another key observation that built on the link between leptin and reproduction is the secretion of leptin from human placenta, further establishing an association between leptin and pregnancy 8, 16. Placental formation during human gestation is crucial for embryonic progress and successful pregnancy outcome, allowing metabolic exchange and production of steroids, hormones, growth factors and cytokines that are critical for the maintenance of pregnancy 17, 18. Trophoblast cells play an essential role in the development of placenta. These cells differentiate into two distinct types: extravillous and villous trophoblast. In the extravillous pathway, cytotrophoblasts (CT) proliferate, differentiate into an invasive phenotype and penetrate into the maternal decidua and myometrium. Meanwhile, in the villous pathway, mononuclear CT fuse to form a specialized multinuclear syncytium called syncytiotrophoblast (ST) 19.

In normal pregnancy, trophoblast invasion is a critical step in remodelling the maternal spiral arteries to adequately perfuse the developing placenta and foetus 20. Failure of invasion processes may lead to miscarriage or pregnancy disorders such as pre‐eclampsia (PE) or intrauterine growth restriction (IUGR) 21, 22. In this sense, deregulation of leptin levels has been implicated in the pathogenesis of various disorders of reproduction and gestation, such as polycystic ovary syndrome (PCOS), recurrent miscarriage, gestational diabetes mellitus (GDM), PE and IUGR 23.

## Leptin mediates the crosstalk between adipose tissue and reproduction

Reproductive function depends on the energy reserves stored in adipose tissue and the reproductive system. The large energy needs for pregnancy was the original rationale to explain the disruption of reproductive function by low fat reserves 24. This led to the hypothesis of an endocrine signal that conveys information to the brain about the size of fat stores 25. Thus, leptin was the first adipokine claimed to be the ‘missing link’ between fat and reproduction 9. Leptin modulates satiety and energy homoeostasis 26, 27, but is also produced by placenta. Thus, it was suggested that the effects of placental leptin on the mother may contribute to endocrine‐mediated alterations in energy balance, such as the mobilization of maternal fat, which occurs during the second half of pregnancy 28, 29. In addition, leptin has been found to influence several reproductive functions, including embryo development and implantation 30. Moreover, animal models have demonstrated that leptin‐deficient mice are infertile, and fertility can be restored by exogenous leptin 31. This adipokine may therefore play a critical role in regulating both energy homoeostasis and the reproductive system 32.

Leptin increases the secretion of gonadotrophin hormones, by acting centrally at the hypothalamus 33. In addition, because leptin has been shown to be influenced by steroid hormones and can stimulate LH release, leptin may act as a permissive factor in the development of puberty 34.

Leptin can also regulate ovary functions 35, 36, 37, 38. Thus, leptin resistance and/or hyperleptinaemia in obesity lead to altered follicle function, whereas conditions in which nutritional status is suboptimal, leptin deficiency results in hypothalamic–pituitary gonadal axis dysfunction 39, 40.

In addition, a significant role of leptin in embryo implantation was proposed. Leptin and leptin receptor are specifically expressed at the blastocyst stage 41, and it was also reported that leptin is present in conditioned media from human blastocysts, promoting embryo development, suggesting a function in the blastocyst–endometrial dialog 42.

## Role of leptin in placenta development

**Figure 1:**
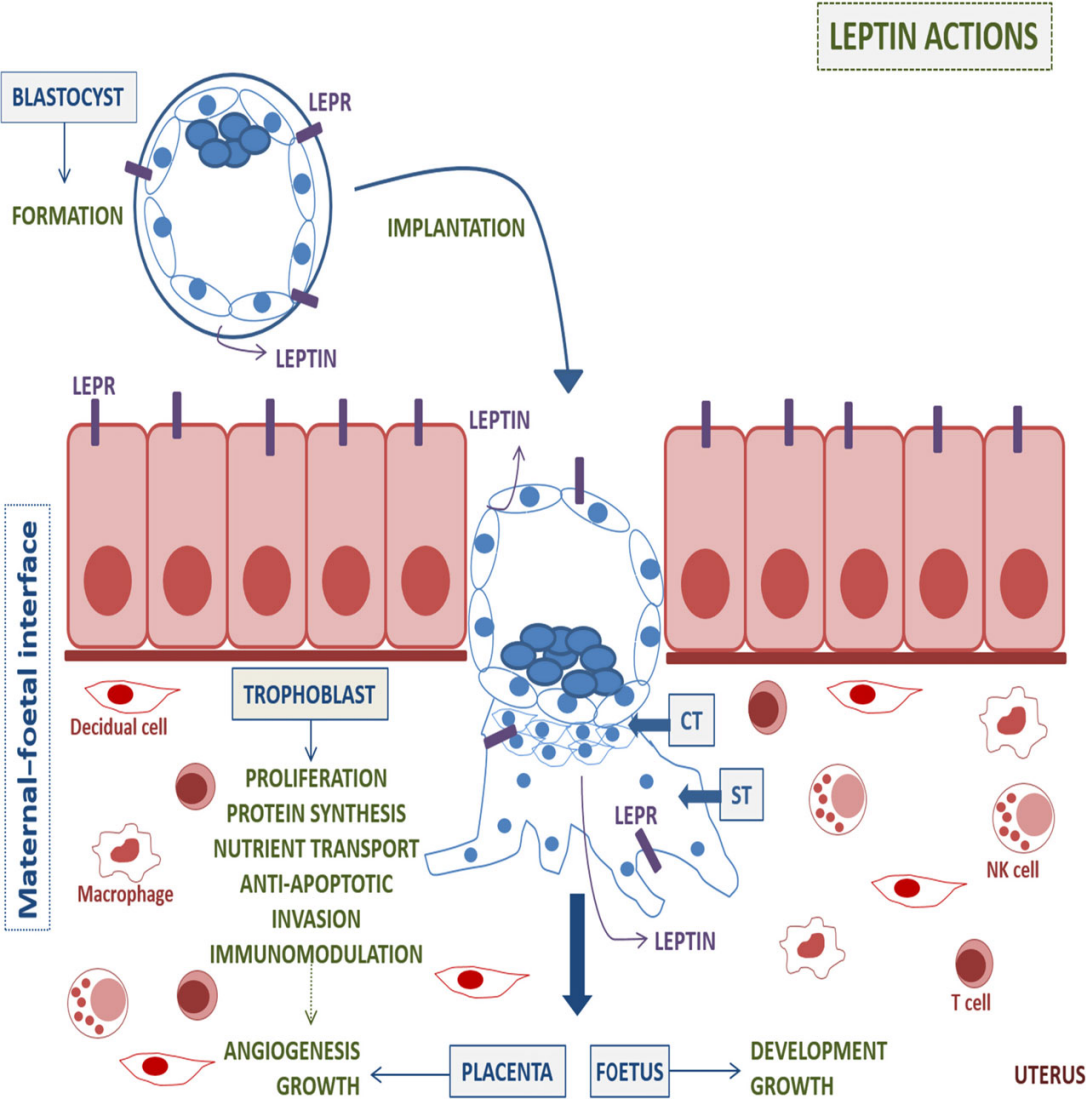
Leptin action during the first stages of pregnancy. Leptin seems to play a crucial role during the first stages of pregnancy as it modulates critical processes such as implantation and placentation, as well as foetus development. The figure summarizes the most important actions of leptin during early gestation (green), highlighting its role in the maternal–foetal interface. It is also shown the different type of cell which expresses leptin receptor (LEPR) and produce leptin (violet). CT: cytotrophoblast, ST: syncytiotrophoblasts.

The implantation process involves complex and synchronized molecular and cellular interplay between the uterus and the implanting embryo, and these events are regulated by paracrine and autocrine factors 18. In 1997, leptin was described as a new placental hormone in humans 29. Circulating leptin levels are significantly increased during pregnancy and decreased after birth, revealing an important role of leptin during gestation 43, 44. Placental production of leptin is one of major source of higher levels in maternal circulating leptin other than maternal gain of fat mass 45. Leptin is now considered an important regulator during the first stages of pregnancy which has physiological effects on the placenta, including angiogenesis, growth and immunomodulation 28, 46, 47, 48, 49, 50, 51. Figure 1 highlights the main actions of leptin in the maternal–foetal interface.

The control of cell proliferation is critical for a correct placental development, and it is finely regulated 52. During placentation, CT and ST keep a subset of cells in direct contact to the villous basement membranes. In the extravillous compartment, cell proliferation favours the invasion of the uterine stroma. Similarly, in the villous compartment cells undergo syncytial fusion directed by specific transcription factors 53. It was shown that leptin induces proliferative activity in many human cell types 54, 55, 56, *via* mitogen‐activated protein kinase (MAPK) activation 57. We have demonstrated that leptin promotes proliferation of trophoblast cells by this MAPK pathway 46, 51 and stimulates protein synthesis by the activation of translation machinery in trophoblastic JEG‐3 cells, a human placenta choriocarcinoma cell line 47, 58.

In this line, multiple signal transduction pathways are activated in response to leptin both in trophoblastic JEG‐3 cell culture and in human term placenta 46. Leptin receptor requires activation of receptor‐associated kinases of the Janus family (JAK) 59. After ligand binding, JAKs autophosphorylate and tyrosine phosphorylate various signal transducers and activators of transcription (STATs). In this context, leptin is able to stimulate Janus kinase (JAK)–STAT pathway by mainly promoting JAK‐2, the most important JAK isoform to mediate physiological effects of leptin 60, and STAT‐3 tyrosine phosphorylation in the human placenta choriocarcinoma JEG‐3 cell line, as well as in trophoblast cells from human term placenta. STAT‐3 activity has been correlated with trophoblast invasiveness 61. In this context, it is interesting to mention the role of Sam68, an RNA‐binding protein originally identified as the substrate of Src kinase during mitosis and a member of the signal transduction and activation of RNA metabolism (STAR) family 62, 63. Leptin stimulates Tyr‐phosphorylation of Sam68 in the trophoblast, mediating the dissociation from RNA, suggesting that leptin signalling could modulate RNA metabolism 64, 65. Moreover, phosphorylated Sam68 interacts with STAT‐3 in response to leptin in trophoblastic JEG‐3 cells 66, suggesting that Sam68 seems to play an important role mediating biological function of leptin 66.

In human trophoblastic cells, it has also been demonstrated that leptin induces the phosphorylation of the extracellular signal‐regulated MEK and the extracellular signal‐regulated ERK 1/2 46. Moreover, it is well‐established that the ERK pathway is essential for reproduction in general, and for the control of trophoblast penetration and invasion 67, as well as placental development 68.

Besides, leptin activation of phosphatidylinositol 3‐kinase (PI3K) pathway has been described in many systems, including placenta 47, 58, leading to phosphorylation of Protein kinase B (PKB), also known as Akt and inactivation of glycogen synthase kinase 3 (GSK‐3), as well as the activation of the translation machinery.

In placental villi, cell turnover is tightly regulated, *via* apoptotic cascade 69. In normal pregnancy, apoptosis is an essential feature of placental development and it is well‐established that trophoblast apoptosis increases with placental growth and advancing gestation 70. Leptin prevents early and late events of apoptosis *via* MAPK pathway 46, 51. The role of leptin was also studied under different stress conditions such as serum deprivation and hyperthermia. Under serum deprivation, leptin increased the anti‐apoptotic B‐cell lymphoma 2 (BCL‐2) protein expression, while down‐regulated the pro‐apoptotic BAX and BH3 interacting domain death agonist (BID) proteins expression as well as caspase‐3 active form and cleaved poly [ADP‐ribose] polymerase 1 (PARP‐1) fragment in Swan‐71 cells, a first‐trimester trophoblast cells isolated from a 7‐week normal placenta 71 and placental explants. In addition, it was demonstrated that p53 and its phosphorylation of serine 46 (Ser‐46), phosphorylation involved in the selectivity of apoptotic target genes, are down‐regulated by leptin suggesting that leptin plays a pivotal role for apoptotic signalling by inhibiting p53 48. Recent studies have demonstrated that MAPK and PI3K pathways are necessaries for this anti‐apoptotic leptin action and it was also demonstrated that murine double‐minute type 2 also known as E3 ubiquitin–protein ligase (MDM‐2) expression is regulated by leptin 49. In placental explants cultured at high temperatures (40°C and 42°C), the extent of Ser‐46 phosphorylation of p53 and the expressions of p53‐regulated apoptosis‐inducing protein 1 (p53AIP1), a potential mediator of apoptosis depending on p53, p21 and Caspase‐3 are increased and these effects are significantly attenuated by leptin, indicating that leptin is a pro‐survival placental cytokine 50. Figure 1 highlights the main actions of leptin in the maternal–foetal interface.

## Leptin as an immunomodulator during pregnancy

One of the most important placental functions is to prevent embryo rejection by the maternal immune system to enable its correct development 72. To ensure normal pregnancy, trophoblast differentiation requires potent immunomodulatory mechanisms to prevent rejection of ST and invasive trophoblast by alloreactive lymphocytes and natural killer (NK) cells present in maternal blood and decidua 73. Inflammatory mediators such as interleukin‐1 β (IL‐1β), interleukin‐6 (IL‐6), tumour necrosis factor α (TNFα) and prostaglandins are produced and secreted by the human placenta and these cytokines play an important role in a number of normal and abnormal inflammatory processes, including the initiation and progression of human labour 74, 75, 76. There are several homologies between the expression and regulation of cytokines and inflammation‐related genes in the placenta and in the white adipose tissue. In this regard, leptin effects include the promotion of inflammation and the modulation of innate and adaptive immunity 64, 77, 78. Thus, placental leptin acts as an immune modulator, regulating the generation of matrix metalloproteinases (MMPs), arachidonic acid products, nitric oxide production and T‐cell cytokines 76. Interestingly, leptin expression is also regulated by interleukin‐1 α (IL‐1α), IL‐1β, IL‐6 and interferon‐ϒ (IFN‐ϒ) 44, 79, 80.

It was reported that leptin stimulates IL‐6 secretion in human trophoblast cells 81, 82. In addition, TNFα release from human placenta is also stimulated by leptin, and it was demonstrated that nuclear transcription factor NF‐kappa B (NF‐ҡB) and peroxisome proliferator‐activated receptor γ (PPAR‐γ) are important mediators of this effect 83. Recently, we have found that leptin induces human leucocyte antigen G (HLA‐G) expression in placenta. HLA‐G has potent immunosuppressive effects promoting apoptosis of activated CD8+ T lymphocytes, the generation of tolerogenic antigen‐presenting cells and the prevention of NK cell‐mediated cytotoxicity. These data place leptin as a placental cytokine which confers to trophoblast cells a tolerogenic phenotype to prevent immunological damage during the first steps of pregnancy 84.

Pro‐inflammatory leptin actions may also have significant implications in the pathogenesis of various disorders associated with pregnancy, such as GDM and PE, which are characterized by increased leptin expression. In this sense, placental leptin may contribute to the incremented circulating levels of pro‐inflammatory mediators that are evident in these pregnancy diseases, whereas successful pregnancy is associated with down‐regulation of intrauterine pro‐inflammatory cytokines 23, 85, 86.

## Leptin and pathologies associated with pregnancy

### Polycystic ovary syndrome

PCOS, the most common endocrine disorder in females and major cause of anovulatory infertility, affects approximately 15% of women during reproductive ages 32. It is characterized by hyperandrogenism, chronic oligoanovulation and polycystic ovarian morphology 87. Peripheral insulin resistance appears to play a crucial role in the pathogenesis of PCOS 88. However, the aetiology of PCOS is not fully understood yet. The deregulated secretion of adipokines, including leptin, plays a role in the pathogenesis of PCOS 89. Besides, PCOS could be associated with increased prevalence of gestational disorders such as miscarriage, GDM and PE 90.

Different studies have shown augmented leptin levels in women with PCOS 91, 92, 93. A recent work confirmed that leptin serum concentrations are increased in obese women with PCOS, while adiponectin levels are decreased 94. Furthermore, the authors suggested the higher leptin levels may be related to the hyperinsulinaemic characteristic of obesity and PCOS 94. In this sense, as women with PCOS also commonly present overweight and obesity 95, the symptoms mentioned could be a consequence of the hyperleptinaemia due to the gain of fat mass. A recent preliminary investigation proposed leptin as strong biomarker of hyperandrogenic PCOS women, suggesting metabolic and inflammatory biomarkers may be increased in PCOS. Interestingly, offspring from PCOS patients have increased inflammatory markers such as matrix metalloproteinase‐9 (MMP‐9) and S100 calcium‐binding protein A8 or calgranulin A (S100A8), suggesting that these children may exhibit increased chronic low‐grade inflammation 96. In fact, it has been reported that increased leptin concentrations may be correlated with insulin resistance, metabolic disorder, infertility and even cardiovascular disease risk in PCOS, which may contribute to the aetiology and development of PCOS 97. Elevated leptin levels could be one of the mechanisms underlying insulin‐mediated ovarian androgen production, as high leptin levels are associated with elevated testosterone levels 98.

### Recurrent miscarriage

Recurrent miscarriage is defined as the loss of three or more consecutives pregnancies before the 20th week of gestation with or without previous live births. Genetic, endocrine, anatomical, immunological, thrombophilic and environmental factors have been implicated in recurrent miscarriage. However, no cause can be found in up to 50% of cases. In patients who have early recurrent miscarriages, some proteins such as human chorionic gonadotrophin (hCG), glycodelin and galectin‐1 are down‐regulated in the ST. Moreover, animal and human studies indicate that alterations in leptin signalling may increase the risk for pregnancy loss 99.

Serum leptin concentration was found elevated in women with recurrent miscarriage in comparison to control group 100. However, in women who subsequently miscarried, it was found that at weeks 5–6 and 7–8 plasma leptin concentrations are also lower than women who subsequently had a term birth 101. In addition, low serum leptin concentrations were observed in women suffering spontaneous miscarriage during the first trimester 102. However, Tommaselli *et al*. did not find significant differences in maternal serum leptin levels, probably due to the heterogeneity of miscarriage in terms of pathogenesis 103.

Single nucleotide polymorphisms (SNP) of LEPR within domains necessary for receptor activation or the cytoplasmic domains may be associated with impaired signalling capacity. In this line, the A223G polymorphism of LEPR is associated with increased risk of pregnancy disorders like PE 104. On the other hand, Chin *et al*. 105 did not find a correlation between this polymorphism and recurrent miscarriage. However, other studies have reported that these genetic variants are associated with pregnancy recurrent loss (Table 1) 100, 106.

**Table 1:** LEP and LEPR single nucleotide polymorphisms (SNPs) in pathologies associated with pregnancy

Pathologies associated with pregnancy	SNP	Description
Polycystic ovary syndrome	–	No LEP or LEPR SNPs have been described
Recurrent miscarriage	LEP‐2548G/A	GA genotype and G allele are associated with risk of RM
Gestational diabetes mellitus	LEP‐2548G/A	A allele is associated with risk of gestational diabetes mellitus (GDM)
Pre‐eclampsia	LEP‐2548G/A	A allele is associated with PE
	LEPR A223G	G allele is associated with increased risk of severe PE
	LEPRG1019A	GA genotype and G allele are associated with severe PE
	LEPR A668G	A allele is associated with severe PE
Intrauterine growth restriction	–	No LEP or LEPR SNPs have been described

### Gestational diabetes mellitus

GDM is the most common pregnancy metabolic disorder and is defined as the type of glucose intolerance that develops in the second trimester and third trimester of the pregnancy, resulting in hyperglycaemia of variable severity 107. Aberrant development and functioning of the placenta, including placental overgrowth, have been implicated as important factors that contribute to GDM‐associated complications 108, 109. GDM is associated with a high perinatal morbidity and mortality as well as insulin resistance, hyperinsulinaemia and hyperleptinaemia, and these GDM‐associated conditions disturb placental nutrient transport and foetal nutrient supply 110, 111. It has been found that leptin and leptin receptor expressions are increased in placenta from GDM 23, 85 and, in fact, leptin was proposed as a first‐trimester biochemical predictor of GDM 112, 113. In addition, it was suggested that hyperinsulinaemia may regulate placental leptin production acting as a circulating signal to control foetal homoeostasis 114, 115. Furthermore, it is though that maternal glucose regulates cord blood leptin levels and this could explain why newborns exposed to GDM have an increased risk of obesity 116. Comparison of the placental gene expression profile between normal and diabetic pregnancies indicates that increased leptin synthesis in GDM is correlated with higher production of pro‐inflammatory cytokines such as IL‐6 and TNFα, causing a chronic inflammatory environment that enhances leptin production 117.

Our group has reported that insulin induces leptin expression in trophoblastic cells by increasing leptin promoter activity 118. It is known that leptin and insulin share several signalling pathways, such as JAK2/STAT‐3, MAPK and PI3K. Moreover, we could demonstrate that in GDM placenta is increased the basal phosphorylation of STAT‐3, MAPK 1/3 and PKB, with resistance to a further stimulation with leptin or insulin *in vitro*, suggesting synergistic interaction and a crosstalk between insulin and leptin signalling in human placenta 23.

On the other hand, GDM is associated with increased incidence of polyhydramnios, due to an increase in amniotic fluid volume, suggesting that aquaporins (AQP), such as AQP9 expression could be altered in GDM 119, 120. Besides, when maternal circulating glucose levels are controlled they have normal amniotic fluid volume. AQP9 is also a transporter for glycerol and may also provide this substrate to the foetus. In this context, we have found that AQP9 messenger RNA (mRNA) and protein expressions are elevated in placentas from women with GDM. These data could suggest that during GDM the overexpression of AQP9, which correlates with higher leptin plasma levels, increments glycerol transport to the foetus and may help to cover the increase in energy needs that occur during this gestational metabolic disorder 121.

### Pre‐eclampsia

PE is a potentially life‐threatening hypertensive disorder affecting ~2–7% of all pregnancies. Approximately 1% of cases are severe, causing stillbirth or the need for extreme preterm delivery. It is characterized by hypertension, systolic blood pressure ≥140 mmHg and/or diastolic blood pressure ≥90 mmHg after 20 weeks of gestation, and proteinuria. Some of the established risk factors are age younger than 20 years or older than 40 years, primiparity, excess bodyweight of the mother, multifoetal pregnancy and familiar and individual history of PE 122. Increasing evidence supports that the pathogenesis of PE involves improper placental development, due to dysfunctional proliferation, migration and invasion of CT into the uterus. This leads to inappropriate spiral artery remodelling, decreased placental blood flow and placental hypoxia 123.

Leptin expression is increased in pre‐eclamptic placentas, and many studies suggest a positive correlation between elevated serum levels and PE 124, 125, 126. Moreover, it has been shown that leptin concentration is higher in term PE but not in preterm PE 126. Thus, leptin has been proposed as a link between body mass index and PE, but the role of obesity or leptin in the pathogenesis of PE is not obvious 126. Leptin up‐regulation could be attributed to placental stress, mainly by the hypoxia present in pre‐eclamptic placenta. Furthermore, serum leptin levels seem elevated in PE even before the clinical onset of the disease, suggesting a possible prognostic value 127. In addition, leptin inhibits increased apoptosis of placental cells during PE. Also, as leptin is a potent angiogenic factor, enhanced placental leptin could increase blood supply to the placenta by neovascularization. Furthermore, leptin is involved in the regulation of placental nutrient transporters, suggesting that hyperleptinaemia in PE is a compensatory response to boost nutrient delivery to the underperfused placenta 117.

However, the role of leptin in PE should be evaluated cautiously as it has recently been found no association of leptin levels with PE 128. SNPs in the LEPR gene have also been investigated in relation to severe PE. In this sense, it was reported that variants of LEPR such as A223G polymorphism may individually modify the risk of severe PE (Table 1) 104.

### Intrauterine growth restriction

The failure of arterial remodelling results in malperfusion of the placenta 129. The incapacity of the placenta to deliver an adequate supply of nutrients to the foetus is termed placental insufficiency and results in IUGR, affecting up to 5–10% of pregnancies in developed countries. IUGR is characterized by a birthweight of <2.5 kg and is associated with a high incidence of perinatal morbidity and mortality and increased risk of cardiovascular and metabolic diseases in adulthood 130. IUGR represents a period of true foetal malnutrition followed by a period of weight recovery after birth, which leads to changes in adipose tissue with important long‐term consequences 131. Specifically, IUGR is frequently associated with inflammation and infarcts within the villi, implying abnormal villous development 132. At the same time, several growth factors and signalling molecules have been implicated in IUGR, including vascular endothelial growth factor and leptin 133.

Diverse studies demonstrated lower circulating leptin levels in IUGR neonates at birth, due to reduced fat mass and/or lower placental production, suggesting leptin as a growth factor that intervenes during foetal intrauterine development 134, 135. Compared with normal birthweight controls, leptin levels become higher in IUGR children and adults, suggesting an adaptive leptin resistance beneficial for catch‐up growth or an adipocyte dysfunction associated with IUGR 136. However, other studies have reported that maternal serum leptin concentrations were significantly higher in pregnancies complicated by foetal growth restriction and growth‐restricted foetuses show umbilical cord leptin concentrations lower than those in normal foetuses, suggesting that it could be due to a compensatory mechanism in which small placentas produce more leptin 137. Simultaneously, it was suggested that placental insufficiency is associated with an increase in placental leptin production 138. Furthermore, some studies demonstrated that leptin levels are lower in IUGR, but differences were not significant 139, 140, and it was also reported that cord blood leptin levels did not differ significantly in IUGR compared to normal pregnancies 141. These data suggest that the association between leptin and IUGR is controversial. Finally, it was reported that the mother of foetuses with growth restriction has a body composition pattern characterized by slightly increased fraction of fat mass and increased serum leptin levels 142.

## Conclusions

In conclusion, it could be affirmed that leptin plays an integral role in the normal physiology of the reproductive system. Leptin controls reproduction depending on the energy state of the body, and sufficient leptin levels are a prerequisite for the maintenance of reproductive capacity. The present review was focused in placental leptin effects during gestation, when leptin levels are increased due to leptin production by trophoblastic cells. Thus, leptin has a wide range of biological functions on trophoblast cells and a role in successful establishment of pregnancy. In this sense, leptin promotes proliferation, protein synthesis and survival of placental cells. These actions are very important as cell proliferation and apoptotic cascades are critical for the correct placental development and function. Moreover, leptin is involved in the promotion of trophoblast invasion which represent a key event during early pregnancy. Besides, it is suggested an important role of leptin in the regulation of immune mechanisms at the maternal interface.

**Figure 2:**
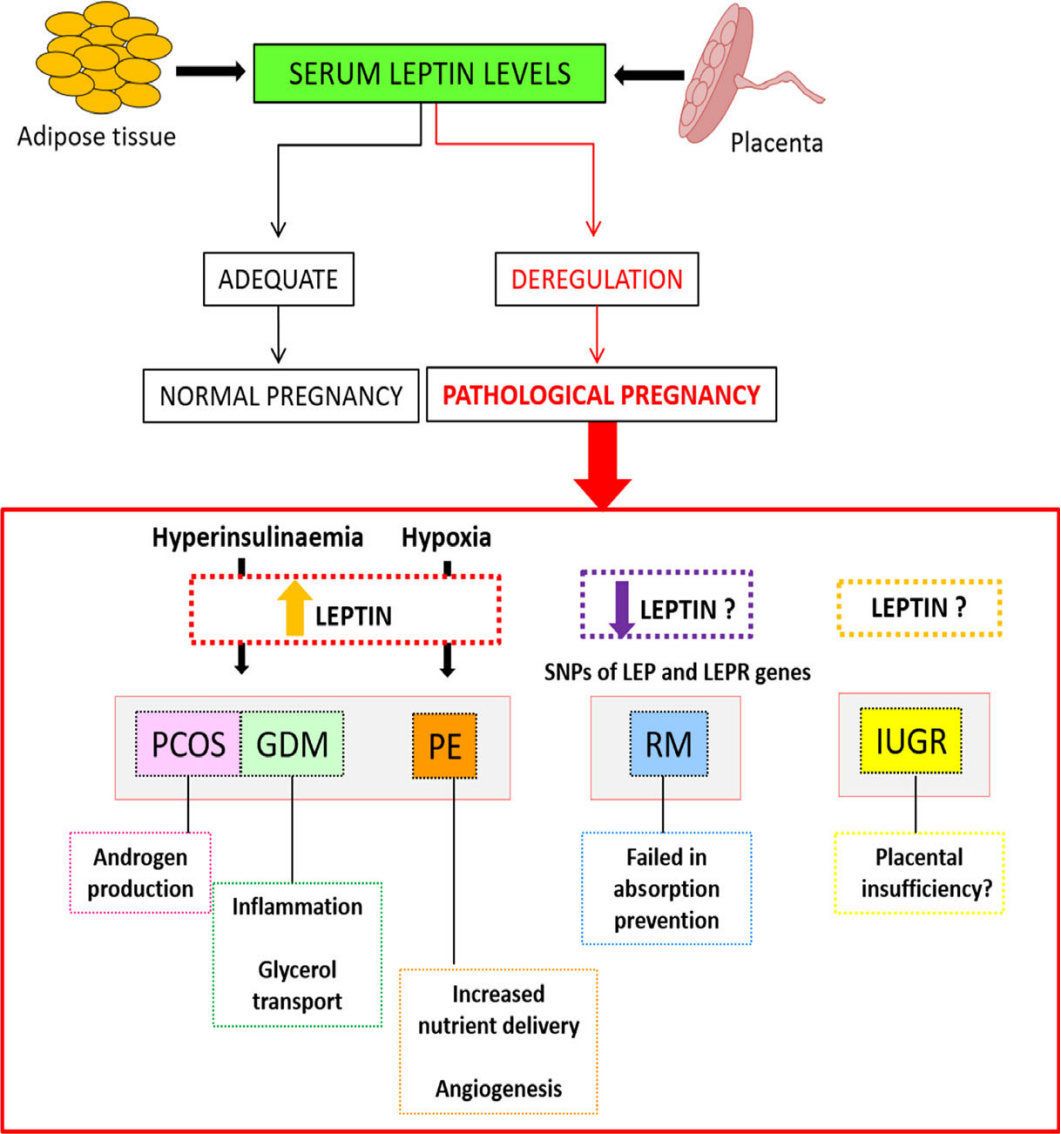
Leptin association pregnancy disorders. Deregulation of leptin levels has been correlated with the pathogenesis of various disorders associated with reproduction and gestation, including polycystic ovary syndrome (PCOS), recurrent miscarriage (RM), gestational diabetes mellitus (GDM), pre‐eclampsia (PE) and intrauterine growth restriction (IUGR). The figure summarizes the link between leptin and the mentioned diseases, including suggested causes and consequences of these pathologies.

On the other hand, observational studies have demonstrated that states of leptin overabundance, deficiency or resistance can be associated with abnormal reproductive function. Clinical studies demonstrate an impact of obesity on the risk of infertility, and it is also established that obesity may lead to deregulation in leptin function that results in maternal disease 143. In this context, leptin deregulation has been implicated in the pathogenesis for at least some disorders associated with reproduction and pregnancy, such as PCOS, recurrent miscarriage, GDM, PE and IUGR. It is well accepted that increased leptin levels are detected in women with PCOS and that may be correlated with insulin resistance, metabolic disorders and infertility. Recurrent miscarriages are associated with altered leptin levels, but the relationship is open for discussion. On the other hand, SNPs of leptin and LEPR genes are risk factors for miscarriage. Leptin and leptin receptor expressions are increased in placentas from GDM, which may be relevant to control foetal homoeostasis. PE is also characterized by enhanced leptin concentrations, even before the clinical onset of the disease, suggesting a possible prognostic significance. Finally, the association between IUGR and leptin levels is controversial. Figure 2 summarizes the link between leptin and the mentioned diseases, including suggested causes and consequences of these pathologies.

Different therapeutic strategies based on leptin administration have been described. Patients with leptin mutations show a marked restoration of fertility as well as weight loss and improvements in immune function after leptin therapy. Furthermore, leptin replacement therapy improves the reproductive abnormalities associated with hypothalamic amenorrhoea (such as failure to menstruate, infertility and premature osteoporosis) 144. On the other hand, compounds that could reverse leptin resistance and act as leptin sensitizers could be beneficial to treat pathologies associated with hyperleptinaemia 145. A number of evidence suggested that leptin might have potential as a treatment for diverse pathologies including the malfunctioning of the reproductive system.

Further investigation is needed to fully elucidate the association of leptin with pathological pregnancy and to establish leptin as a biomarker for pathologies associated with pregnancy.

## Conflict of interest

The authors declare no conflict of interest.
